# Adenosine triphosphate as a modulator of protein interactions and stability

**DOI:** 10.1002/2211-5463.70312

**Published:** 2026-07-28

**Authors:** Shuyuan Tan, Robin Curtis

**Affiliations:** ^1^ Department of Chemical Engineering University of Manchester UK

**Keywords:** amyloid formation, ATP, colloidal stability, phase separation, protein aggregation, protein–protein interactions

## Abstract

Beyond its classical role as the cellular energy currency, adenosine triphosphate (ATP) can directly modulate protein stability, solubility and self‐assembly through weak nonspecific interactions. Here, we summarise the current experimental and theoretical understanding of noncanonical ATP–protein interactions and their implications for folding, aggregation and phase stability. Recent studies show that ATP interacts primarily with positively charged residues such as lysine (Lys) and arginine (Arg) via electrostatic and hydrogen‐bonding interactions, with additional cation–π or π–π contacts in some systems. These interactions modulate protein folding, conformational stability and aggregation by shifting the balance between folded and unfolded states. For intrinsically disordered and amyloidogenic proteins, ATP acts as a concentration‐dependent regulator that can promote, suppress or remodel phase‐separated condensates and fibrillar assemblies depending on protein sequence, ATP concentration and solution conditions. In natively folded proteins, ATP influences protein–protein interactions and phase behaviour, often enhancing colloidal stability and suppressing aggregation. Complexation with Mg^2+^ further modulates these effects by altering phosphate charge accessibility and, in some systems, changing the balance between binding, bridging and solubilisation. Together, these observations establish ATP as a multivalent small‐molecule modulator of protein behaviour.

## Graphical abstract

ATP is best known as the cell's energy currency, but it also shapes how proteins fold, interact, aggregate and form biomolecular condensates. This review explains the emerging physical principles behind these effects, including weak binding to charged protein regions, magnesium‐dependent behaviour and concentration‐dependent control of protein stability, solubility, phase separation and amyloid formation across diverse biomacromolecular systems.

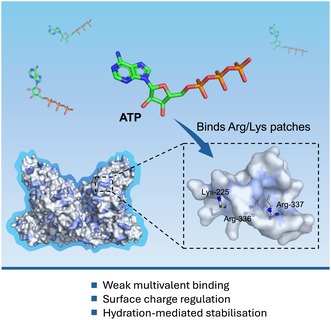

**Abbreviations**
ADP: adenosine diphosphate
AMP: adenosine monophosphate
ATP: adenosine triphosphate
Aβ: amyloid‐β
BSA: bovine serum albumin
C71G‐hPFN1: C71G mutant human profilin‐1
CAPRIN1: cytoplasmic activation/proliferation‐associated protein 1
CSP: chemical shift perturbation
CTP: cytidine triphosphate
DOSY: diffusion ordered spectroscopy
FGF2: fibroblast growth factor 2
FUS: fused in sarcoma
GTP: guanosine triphosphate
hIAPP: human islet amyloid polypeptide
HSA: human serum albumin
hSOD1: human superoxide dismutase 1
HSQC: heteronuclear single quantum coherence
IDP: intrinsically disordered protein
IDR: intrinsically disordered region
LLPS: liquid–liquid phase separation
mAb: monoclonal antibody
Mg–ATP: magnesium–ATP complex
Mot3‐PrD: prion domain of the yeast transcription factor Mot3
NAC: nonamyloid‐β component
NGF: nerve growth factor
NMR: nuclear magnetic resonance
NOE: nuclear Overhauser effect
PLD: prion‐like domain
polyP: inorganic polyphosphate
PP: pyrophosphate
PRE: paramagnetic relaxation enhancement
RNAseA: ribonuclease A
RRM: RNA recognition motif
STD: saturation transfer difference
Sup35 NM: N‐terminal and middle domains of yeast Sup35
SYNCRIP: synaptotagmin‐binding cytoplasmic RNA‐interacting protein
TDP‐43: TAR DNA‐binding protein 43
T_m_
: melting temperature
TPP: tripolyphosphate
UTP: uridine triphosphate

## ATP beyond its biological roles

Adenosine triphosphate (ATP) is conventionally recognised as the universal ‘energy currency’ of the cell, driving a vast range of enzymatic and physiological processes including biosynthesis, active transport, signalling and muscle contraction. However, this traditional perspective does not fully explain why intracellular ATP concentrations, typically in the range of 1–10 mm, substantially exceed those required to saturate most ATP‐dependent enzymes [1, 2]. This discrepancy has led to the proposal that ATP possesses additional noncanonical functions beyond its role as a metabolic energy donor [3]. Consistent with this idea, reduced intracellular ATP levels are strongly associated with diseases involving protein aggregation and amyloid deposition [4, 5, 6, 7, 8]. ATP depletion, whether induced genetically or chemically, promotes cytotoxic protein aggregation and condensation *in vivo* [4, 5]. More broadly, ATP concentration has been linked to bacterial persistence, antibiotic tolerance and stress responses in both bacteria and yeast, where reduced ATP levels trigger widespread protein condensation and precipitation, while restoration of ATP promotes disaggregation and recovery of protein solubility [9, 10, 11]. ATP depletion in mammalian neurons promotes pathological protein condensation, while restoration of ATP reverses aggregation and restores cytosolic fluidity [12]. Although some of these effects arise from energy‐dependent cellular processes, growing evidence indicates that ATP can also directly modulate protein assembly through weak, nonspecific interactions, as first proposed by Patel et al. [3]. Supporting this interpretation, Sridharan et al. showed that ATP enhances the solubility of a substantial fraction of the otherwise insoluble proteome [13], while Zuo et al. demonstrated that ATP depletion increases nonspecific electrostatic and hydrophobic associations between cytoplasmic components [14]. In parallel, a growing body of work has explored how noncanonical ATP binding influences protein folding stability [15, 16], aggregation and amyloid formation [3, 17, 18, 19, 20, 21, 22, 23], and liquid–liquid phase separation (LLPS) [3, 24, 25, 26, 27, 28, 29, 30, 31]. Interestingly, weak nonspecific ATP interactions have also been proposed to facilitate enzymatic function by enhancing ATP transport to catalytic sites [32, 33]. Together, these observations have shifted the perception of ATP from a simple metabolic substrate to a broader physicochemical regulator of protein behaviour.

**Fig. 1:**
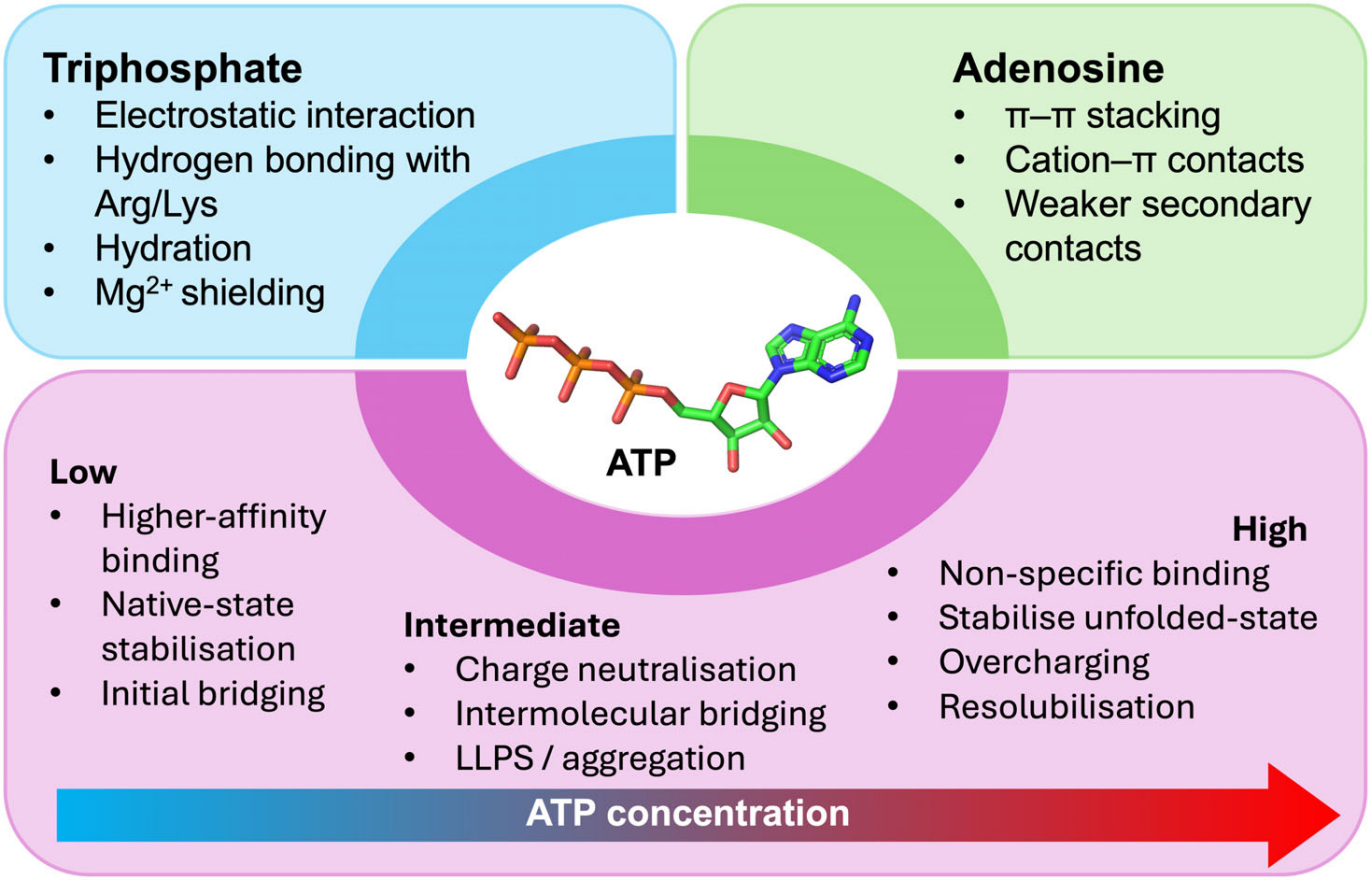
Conceptual framework for adenosine triphosphate (ATP)‐mediated modulation of protein stability and phase behaviour. The triphosphate group provides the dominant electrostatic, arginine/lysine (Arg/Lys)‐associated and hydration‐mediated contributions, whereas the adenosine moiety can add weaker secondary contacts. The concentration regimes shown are representative of systems in which ATP binding is dominated by positively charged protein regions: at low ATP concentrations, high‐affinity binding promotes stabilisation of the native state; at intermediate ATP concentrations, charge neutralisation and intermolecular bridging can occur, particularly in cationic or Arg/Lys‐rich systems; and at high ATP concentrations, non‐specific binding promotes unfolded‐state stabilisation, overcharging or resolubilisation. The overall outcome is governed by the protein sequence, charge state, conformational ensemble, aggregation pathway and solution conditions.

Several recent reviews have addressed different aspects of noncanonical ATP–protein interactions. Song and coworkers focussed mainly on ATP interactions with nucleic‐acid‐binding proteins associated with LLPS [26, 34], while Greiner and coworkers emphasised the role of ATP in age‐related eye disease and protein aggregation in the lens [8, 35]. Paoletti et al. reviewed ATP interactions in nerve growth factor (NGF) and proNGF systems [36]. Most closely related to the present work, Hautke and Ebbinghaus surveyed the emerging role of ATP as a modulator of protein folding, aggregation, phase separation and biomolecular solubility through weak nonspecific interactions [37]. Building on these studies, the present review provides a broader comparative perspective on ATP‐mediated self‐assembly in both globular and intrinsically disordered proteins. The first part focuses on experimental insight into the nature of noncanonical ATP‐binding sites obtained from techniques such as NMR and model compound studies, while the second part examines how these interactions influence self‐assembly processes including phase separation, aggregation and amyloid fibril formation. These structural and concentration‐dependent regimes are summarised schematically in Fig. 1.

## Molecular basis for ATP binding to proteins

### Binding to positively charged sites

High‐resolution NMR studies have provided detailed insight into the nature of noncanonical ATP‐binding sites across a range of proteins. For example, residue‐resolved heteronuclear single quantum coherence (HSQC) NMR measurements have revealed ATP interactions with both intrinsically disordered proteins, such as α‐synuclein, and folded proteins including ubiquitin and the dimeric ubiquitin‐binding domain of p62 [38]. Similarly, Paoletti et al. identified two ATP‐binding sites located on solvent‐exposed loop regions of NGF [39]. In lysozyme, six ATP‐binding sites were identified, one of which overlaps with the canonical nucleic‐acid‐binding region and three of these sites were corroborated by crystallographic identification of bound ATP [40]. Song and coworkers further showed by NMR that ATP binds a range of RNA‐ and nucleic‐acid‐binding proteins, including the RNA recognition motifs (RRMs) of fused in sarcoma (FUS) protein and TAR DNA‐binding protein 43 (TDP‐43) [41, 42], the low‐complexity Arg/Lys‐containing intrinsically disordered regions (IDRs) of FUS and TDP‐43 [24, 43, 44], the acidic nucleic‐acid‐binding domain of synaptotagmin‐binding cytoplasmic RNA‐interacting protein (SYNCRIP) [45] and the folded RNA‐binding domains and IDRs of the SARS‐CoV‐2 nucleocapsid protein [46, 47]. In these systems, ATP primarily targets canonical nucleic‐acid‐binding pockets or low‐complexity Arg/Lys‐rich regions that normally mediate RNA or DNA interactions. Collectively, these studies show that nonspecific ATP binding occurs primarily at weak, solvent‐exposed, positively charged surface regions enriched in arginine and lysine residues, with affinities typically in the millimolar range [26, 39, 40]. This behaviour contrasts with biological ATP‐binding sites, which exhibit substantially higher affinities due to specific hydrogen bonding, electrostatic complementarity and well‐defined structural motifs such as deep binding clefts or canonical nucleotide‐binding regions [48, 49, 50].

A notable and consistent feature of these nonspecific binding sites is their pronounced preference for arginine over lysine. In lysozyme, ATP binding occurs predominantly at arginine‐containing sites and, at elevated ATP concentrations, extends to nearly all arginine residues while remaining comparatively limited for lysine residues [40]. Similar trends are observed in intrinsically disordered proteins. In TDP‐43, ATP binds preferentially to arginine‐rich regions and biphasically modulates LLPS, whereas substitution of arginine with lysine weakens ATP binding and abolishes LLPS [43]. Likewise, NMR studies of the SARS‐CoV‐2 nucleocapsid protein showed that ATP binding within the intrinsically disordered regions is dominated by arginine residues, despite the presence of lysines [47]. Consistent behaviour is also observed for polyphosphate interactions, where replacement of arginine with lysine in histatin‐5 reduces tripolyphosphate (TPP)‐induced phase separation [51]. The stronger interaction of arginine with phosphate groups arises from the guanidinium side chain, which can form multidentate hydrogen‐bonding and more delocalised electrostatic interactions with phosphate moieties than lysine [52, 53].

Multiple studies indicate that ATP and TPP interact with protein surfaces in a highly similar manner, implying that binding is dominated primarily by the multivalent triphosphate group. In lysozyme, TPP binds to the same nonspecific surface sites as ATP with comparable millimolar affinities, although binding to the canonical nucleic‐acid‐binding region is significantly weaker in the absence of the adenosine moiety [40]. Similarly, ζ‐potential measurements for lysozyme, α‐chymotrypsinogen, bovine and human serum albumin (BSA and HSA, respectively), and related proteins show nearly identical profiles for ATP and TPP, indicating comparable charge neutralisation and surface association [40, 54, 55]. However, several studies also indicate that the adenosine moiety can contribute additional short‐range interactions that enhance specificity towards arginine‐rich regions [56, 57]. In particular, Alshareedah et al. showed that poly(A) binds substantially more strongly to arginine‐rich peptides than to lysine‐rich analogues, whereas interactions with polyphosphate alone were similar for both peptide types [56].

### Binding to conformationally flexible and solvent‐exposed regions

ATP binding is generally weak and nonspecific, but its magnitude depends strongly on protein dynamics and solvent accessibility. In intrinsically disordered proteins such as α‐synuclein, ATP interactions are enhanced by the high degree of conformational flexibility, whereas in folded proteins such as ubiquitin and p62, binding is localised primarily to flexible, solvent‐exposed loops and termini rather than rigid structural cores [38]. Molecular dynamics simulations similarly show preferential ATP accumulation at dynamically labile surface regions in proteins including lysozyme, ubiquitin and malate dehydrogenase [58]. However, flexibility alone is not sufficient to define ATP‐binding sites. For example, despite its intrinsic mobility, the interdomain loop region of lysozyme shows no detectable ATP binding by NMR, whereas other flexible surface regions, including the C terminus, do exhibit ATP association [40]. Together, these observations indicate that ATP binding is favoured by conformational flexibility and solvent exposure, but also depends on the local distribution of positively charged residues.

### Binding to nonpolar regions and aromatics

The frequently cited view of ATP as a ‘biological hydrotrope’ implies that, beyond binding to positively charged regions, ATP can associate with exposed non‐polar surfaces on proteins. This interpretation is largely based on observations that ATP, but not TPP, can resolubilise phase‐separated FUS and suppress amyloid formation of Aβ42 [3, 17]. In these systems, the adenosine moiety has been proposed to form transient interactions with hydrophobic or aromatic residues, while the highly charged triphosphate group maintains solvation and limits irreversible aggregation. Consistent with this, ATP has been shown to solubilise hydrophobic probe molecules such as fluorescein diacetate and to alter the emission of ANS, indicating the formation of less polar microenvironments [3].

However, it is unlikely that these effects arise from direct interactions with aliphatic, nonpolar groups in a manner analogous to classical amphiphilic hydrotropes. In particular, Mehringer et al. showed that ATP does not lower the surface tension of water nor enhance the solubility of hydrophobic dyes, in contrast to classical hydrotropes such as sodium xylene sulfonate (SXS) [17]. Instead, ATP behaves similar to inorganic polyphosphates, displaying salting‐out behaviour consistent with its position on the kosmotropic side of the Hofmeister series. This interpretation is further supported by the observation that ATP and TPP exhibit nearly identical effects on the solubility of nonpolar molecules and on the melting temperature T_m_ of proteins such as lysozyme, BSA, conalbumin and ovalbumin [17]. As the unfolded state of these proteins exposes hydrophobic residues, the similarity in T_m_ shifts suggests that the adenosine moiety contributes minimally to interactions with nonpolar groups. This conclusion is reinforced by studies on neutral model macromolecules such as poly(N‐isopropylacrylamide) (PNIPAM), which contains an aliphatic backbone [59]. In this system, ATP, adenosine monophosphate (AMP) and tripolyphosphate all induce a decrease in the lower critical solution temperature, indicative of salting‐out behaviour, whereas adenine and adenosine have negligible impact on the phase transition.

ATP interactions with aromatic residues are distinct from interactions with aliphatic nonpolar groups because aromatic side chains provide an additional capacity for π–π interactions with the adenine ring. Indeed, molecular simulation studies of amyloidogenic peptides and aromatic‐rich IDRs increasingly support a role for direct ATP–aromatic interactions, particularly through the adenosine moiety [17, 31, 60, 61, 62, 63, 64]. Direct experimental evidence for ATP interactions with aromatic systems is provided by a fluorescence spectroscopy study indicating nucleotides preferentially bind to pyrene, a model aromatic probe [65]. However, experimental evidence for such interactions in protein systems is less straightforward and depends strongly on both the protein system and the ATP concentration regime. At low ATP concentrations, typically in the submillimolar to low millimolar range, NMR chemical shift perturbation (CSP) experiments consistently show that ATP binding is dominated by electrostatic interactions with Arg/Lys residues, with little or no detectable involvement of aromatic residues. Kang et al. [24] used HSQC NMR to study ATP binding to FUS and found no detectable interactions with the aromatic‐rich prion‐like domain (PLD), despite clear binding to Arg/Lys‐rich regions. Similarly, in α‐synuclein, Kamski‐Hennekam et al. [18] employed residue‐resolved HSQC NMR to map ATP binding and showed that chemical shift perturbations are largest in the Lys/Thr‐rich N‐terminal region, while the NAC and C‐terminal regions, which contain aromatic residues, exhibit comparatively weak perturbations. In contrast, in the cytoplasmic activation/proliferation‐associated protein 1 (CAPRIN1) system, paramagnetic relaxation enhancement (PRE) and intermolecular nuclear Overhauser effect (NOE) measurements show that ATP binds predominantly to Arg residues at low ATP concentrations (∼1 mm), whereas at higher concentrations (∼50 mm), additional contacts involving aromatic residues become apparent [25, 27]. Related studies from Song and coworkers further support the dominance of ATP interactions with positively charged residues over aromatic interactions [26, 42, 45]. In the RRM domains of TDP‐43 and the noncanonical RNA‐binding acidic domain of SYNCRIP, ATP binds preferentially to positively charged nucleic‐acid‐binding surfaces that also contain aromatic residues. NMR and docking analyses showed that the triphosphate group forms the dominant electrostatic and hydrogen‐bonding interactions with Arg/Lys‐rich regions, whereas interactions involving the adenine ring are comparatively weaker. A similar conclusion can be drawn from the observation that ATP binds to positively charged surface sites on folded proteins with affinities comparable to those measured for nucleic‐acid‐binding domains containing both aromatic and basic residues [39, 40]. Representative systems illustrating these triphosphate‐dominated and adenosine‐assisted contributions are summarised in Table 1.

**Table 1:** Representative evidence distinguishing triphosphate‐dominated and adenosine‐assisted contributions to non‐canonical ATP–protein interactions.

System / context	Evidence and comparators	Main mechanistic implication	References
Proteome‐wide cellular systems	ATP depletion, restoration, proteome solubility profiling, and intracellular associative‐interaction probes.	ATP availability is linked to proteome solubility and cellular material state, although direct cosolute effects remain difficult to separate from ATP‐dependent cellular processes.	[4, 9, 10, 13, 14]
Serum albumins (HSA/BSA)	DOSY NMR, binding assays, ζ‐potential, and thermal aggregation; ATP compared with TPP and related nucleotide/phosphate species.	Albumins show both nucleotide‐like binding and weaker distributed interactions; ATP–TPP similarities support a major triphosphate‐dominated electrostatic contribution.	[54, 55, 66, 67, 68]
Cationic folded proteins	Phase behaviour, NMR, and ζ‐potential; ATP and TPP compared with salt and Mg^2+^.	For positively charged proteins, ATP and TPP can drive charge neutralisation, bridging, reentrant phase behaviour, and excess‐anion resolubilisation.	[30, 40, 69]
Solvent‐exposed and flexible protein regions	Residue‐resolved NMR and molecular simulations on folded and disordered proteins.	ATP binding is often weak, diffuse, and enriched near solvent‐exposed or dynamically labile regions, but flexibility alone does not define binding specificity.	[38, 57, 58]
Basic IDRs and condensate‐forming proteins	NMR, mutagenesis, LLPS assays, and electrostatic‐potential mapping; ATP compared with nucleic acids, TPP, and polyphosphates.	Arg/Lys–phosphate interactions provide a dominant driving force for condensation, bridging, and resolubilisation; adenosine‐associated contacts can add system‐dependent specificity.	[24, 28, 43, 44, 51]
Hydrotrope versus Hofmeister behaviour	Surface tension, hydrophobic‐solute assays, thermal stability, and ATP comparisons with TPP, classical hydrotropes, and neutral cosolutes.	ATP should not be treated as a simple hydrotrope: its effects reflect combined triphosphate hydration, electrostatics, Hofmeister‐type behaviour, and adenosine‐associated contacts.	[3, 17, 37, 59, 65]
Aggregation‐prone, amyloidogenic, and unfolded states	Aggregation assays, NMR, dynamic light scattering, fluorescence, native gels, and refolding assays; ATP compared with Mg–ATP, ADP/AMP, TPP, adenosine, and polyP.	ATP can suppress, remodel, or promote aggregation depending on sequence, charge state, pH, aggregation stage, and whether triphosphate‐mediated bridging or hydration dominates.	[18, 22, 23, 29, 60, 70, 71, 72]

## Insights into ATP binding from protein conformational changes

The effects of additives on protein conformational stability can be understood within the preferential interaction framework developed by Timasheff [73]. In this framework, additives influence protein stability through differences in their interactions with the folded and disordered conformational ensembles. As the additive concentration increases, the conformational state exhibiting the greater net preferential binding with the additive becomes thermodynamically favoured. Experimentally, these effects are commonly assessed through measurements of T_m_, where an increase in T_m_ indicates stabilisation of the folded state, while a decrease reflects destabilisation.

Combining T_m_ measurements with ATP‐binding studies provides insight into how ATP interactions differ between folded and unfolded protein states. In serum albumins, early studies identified a high‐affinity ATP‐binding site on BSA with submillimolar affinity [66, 67], whereas later diffusion ordered spectroscopy (DOSY) NMR, ζ‐potential and saturation transfer difference (STD) NMR measurements showed that both BSA and HSA also possess a larger number of weaker ATP‐ and TPP‐binding sites (*n* ≈ 10) with millimolar affinities [54, 68]. Concomitantly, at low ATP concentrations, T_m_ increases, consistent with stabilisation through occupation of a higher‐affinity native‐state binding site, whereas at higher concentrations T_m_ decreases, indicating that the weaker nonspecific binding interactions become more favourable in partially unfolded or unfolded states. Similar behaviour is observed for lysozyme, where ATP increases T_m_ at submillimolar concentrations [58], but decreases it at higher concentrations [17].

Proteome‐wide measurements provide further insight into the relationship between nucleotide binding, conformational stability and solubility. Using combined thermal proteome profiling and solubility proteome profiling, Sridharan et al. showed that proteins with biological ATP‐binding sites exhibit increases in T_m_ at submillimolar ATP concentrations, consistent with stabilisation through specific ligand binding [13]. At higher ATP concentrations (∼10 mm), however, ATP solubilised a substantial fraction of the positively charged insoluble proteome while simultaneously decreasing T_m_. This contrasts with strong biological binding interactions, which are typically lost upon unfolding due to disruption of well‐defined binding pockets. Importantly, enhanced binding to partially unfolded states can simultaneously increase their solubility, indicating that ATP can exhibit chaperone‐like behaviour by stabilising soluble non‐native conformations and suppressing irreversible aggregation. This behaviour is analogous to inorganic polyphosphates, which act as primordial chaperones by preferentially interacting with unfolded or partially unfolded proteins, maintaining them in soluble, refolding‐competent states and preventing irreversible aggregation [70, 71].

In the proteome‐wide study, guanosine triphosphate (GTP) produced analogous effects for a subset of proteins, including apparent solubility‐related changes, suggesting that weak nucleotide‐mediated solubilisation is not restricted to ATP or to the adenine nucleobase [13]. This interpretation is consistent with residue‐resolved NMR measurements by Nishizawa et al., who found that GTP, cytidine triphosphate (CTP) and uridine triphosphate (UTP) produced ATP‐like chemical‐shift perturbations in α‐synuclein, indicating broadly similar weak interactions for several nucleoside triphosphates [38]. Similarly, Hasche et al. showed that ATP, GTP, CTP and UTP can all bind NGF, further supporting the idea that noncanonical protein binding is not unique to ATP [74]. However, differences between nucleobases can still modulate solubilising efficiency. Experimentally, Pandey et al. showed using pyrene fluorescence that GTP provides a less polar microenvironment for the model aromatic compound pyrene at lower concentrations than ATP, indicating stronger hydrotropic activity for GTP [65].

Additional studies further demonstrate that ATP binding can modulate protein conformational equilibria, activity and stability in diverse ways depending on the protein system and interaction regime. For example, Gnutt et al. showed that weak quinary interactions involving ATP and other cellular components partially destabilise human superoxide dismutase 1 (hSOD1) in cells, with energetic contributions on the order of ∼1 kcal·mol^−1^ [16]. In contrast, Bharadwaj et al. reported that ATP, adenosine diphosphate (ADP) and AMP bind cytochrome C with increasing affinity in the order AMP < ADP < ATP, producing conformational changes that enhanced peroxidase activity by up to ∼6.5‐fold while simultaneously increasing resistance to oxidative and chaotropic stress [75, 76]. Similarly, Rose showed that ATP binds fibroblast growth factor 2 (FGF2) with a dissociation constant of ∼60 μm, induces detectable shifts towards increased random‐coil content and markedly stabilises the protein against degradation in aqueous solution [77]. Notably, the stabilising effect increased with phosphate number, with ATP producing substantially larger effects than ADP or AMP. Related effects have also been reported for the ATP‐independent molecular chaperone α‐crystallin, where ATP binding at low millimolar concentrations induces compaction, increases exposure of hydrophobic sites, enhances substrate association and improves chaperone efficiency [72]. Consistent trends were also reported by Kang et al. for conformationally labile systems such as C71G mutant human profilin‐1 (C71G‐hPFN1) and nascent hSOD1, where ATP and TPP exhibited comparable ability to shift the equilibrium towards folded states, ADP was less effective, and AMP showed little or no folding‐inducing capacity [78]. However, despite its similar folding‐inducing capacity, free TPP strongly promoted aggregation, whereas ATP maintained protein solubility, indicating that the adenosine moiety can modulate the balance between folding and aggregation. A similar phosphate‐number dependence was observed for ATP binding to a monoclonal antibody (mAb) and NGF: Tian and Qian found that ATP, but not ADP or AMP, produced a strong static interaction with a mAb [30], while Hasche et al. reported stronger NGF binding for ATP than ADP, with little or no detectable binding for AMP [74]. Together, these studies indicate that non‐canonical nucleotide–protein interactions are often governed primarily by the number of phosphate groups.

## ATP binding modulated by magnesium

Under cellular conditions, ATP is present at millimolar concentrations, typically ∼1–10 mm, and ATP speciation is strongly coupled to intracellular Mg^2+^, with Mg–ATP expected to represent the dominant physiological form of ATP [1, 79, 80]. Estimates that more than 90% of intracellular ATP is Mg^2+^‐complexed imply that noncanonical ATP–protein interactions *in vivo* are likely to involve a mixture of Mg–ATP, free ATP, free Mg^2+^ and competing cellular ligands, rather than ATP alone [31, 80]. Because ATP binding is dominated by electrostatic interactions involving the triphosphate group, Mg^2+^ complexation would be expected to attenuate binding by shielding phosphate charge; however, the magnitude and even direction of this effect appear to be highly system‐dependent. For lysozyme, Zalar et al. showed by NMR that equimolar Mg–ATP binds to the same six surface sites as ATP, with similar apparent affinities, indicating that Mg^2+^ complexation does not substantially alter the local binding‐site distribution or strength [40]. This contrasts with Nishizawa et al., who found that NaCl and MgCl_2_ generally weaken ATP–protein interactions through electrostatic screening or Mg–ATP formation, although broadly similar binding regions were retained [38]. Notably, in α‐synuclein, inclusion of Mg^2+^ also produced perturbations in acidic regions, consistent with a model in which Mg^2+^ can bridge ATP to acidic protein sites [38]. Kamski‐Hennekam et al. reached a related conclusion for α‐synuclein: Mg–ATP interacted with the same N‐terminal Lys/Thr‐rich region as ATP, but more weakly, while ATP also sequestered Mg^2+^ away from the acidic C‐terminal region [18]. A weakening effect of Mg^2+^ is also consistent with the fluorescence‐quenching study of Tian and Qian, where MgCl_2_ disrupted the static ATP–mAb interaction more effectively than NaCl, attributed to formation of an [Mg–ATP]^−^ chelate with reduced negative charge density [30]. Similarly, Calamai et al. showed that ATP bound tightly to preformed acylphosphatase and lysozyme amyloid fibrils, but was released upon addition of MgSO_4_, supporting a reversible, electrostatically dominated ATP–amyloid interaction [81]. On the other hand, Mg^2+^ does not always suppress ATP association. In CAPRIN1, Mg–ATP binds preferentially to Arg‐rich positively charged regions, which is broadly consistent with the Mg^2+^‐free ATP electrostatic‐potential changes observed by Toyama et al. [25, 27]. Mahapatra et al. further showed that Mg^2+^ can enable or strengthen ATP‐mediated effects in positively charged amyloidogenic systems: ATP promoted phase separation of FUS and aggregation of the N terminus and middle domains of yeast Sup35 (Sup35 NM) only in the presence of Mg^2+^, and Raman spectra of Sup35 NM amyloids indicated weaker ATP association when fibrils were formed without Mg^2+^ [82].

## Impact on protein phase separation

**Fig. 2:**
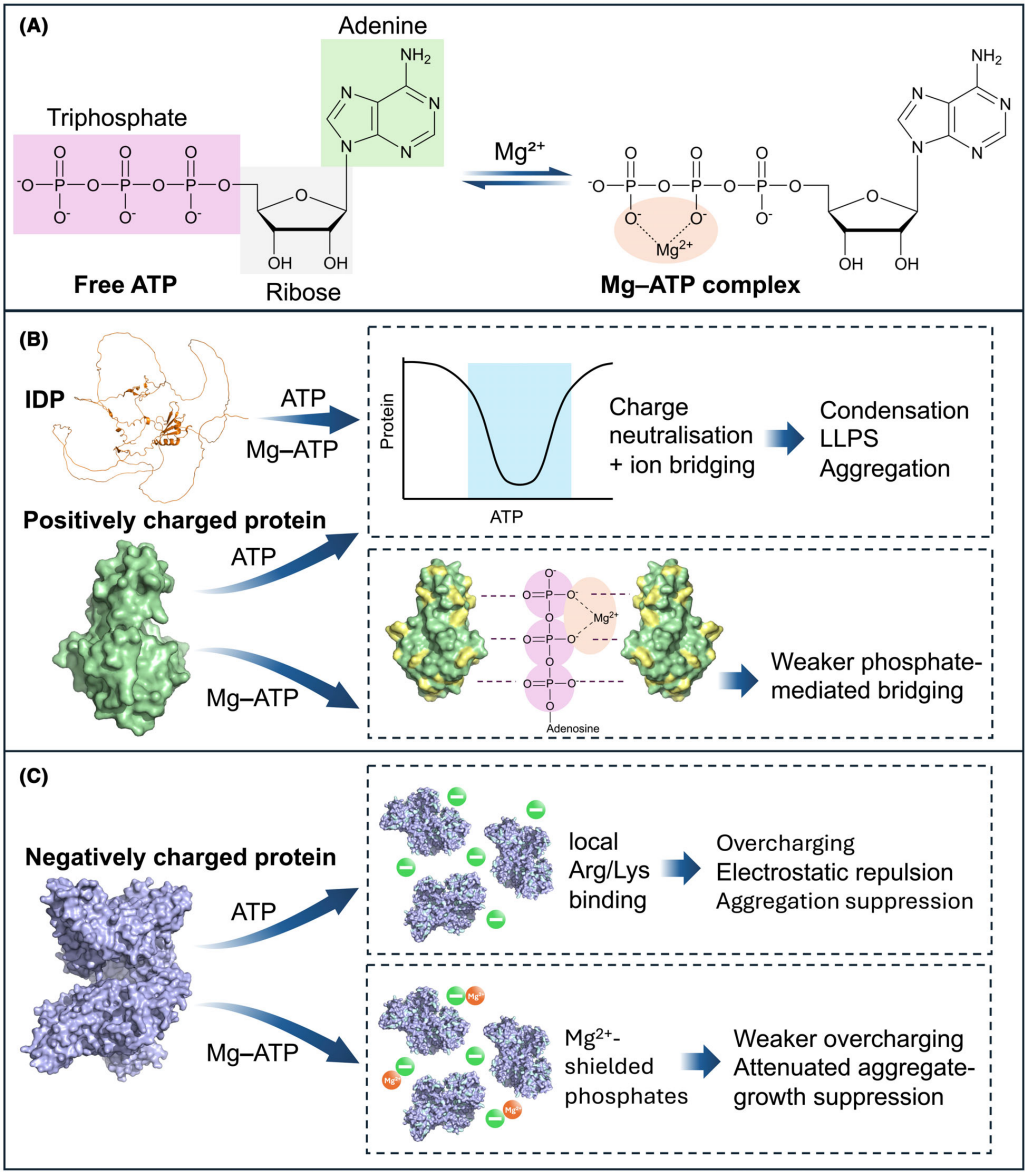
Adenosine triphosphate (ATP) speciation and protein charge context regulate ATP‐mediated protein interactions. (A) Free ATP presents an exposed triphosphate group, whereas magnesium ion (Mg^2+^) coordination forms magnesium–ATP (Mg–ATP) and alters phosphate accessibility. (B) For positively charged proteins, and similarly for some arginine/lysine (Arg/Lys)‐rich intrinsically disordered proteins (IDPs), free ATP can promote charge neutralisation, phosphate‐mediated bridging, liquid–liquid phase separation (LLPS), condensation or aggregation. In many positively charged protein systems, Mg–ATP attenuates these bridging effects, whereas effects in IDPs may be more system‐dependent. (C) For negatively charged proteins, ATP can still bind to local Arg/Lys‐rich patches, promoting overcharging, electrostatic repulsion, and suppression of aggregate growth, whereas Mg^2+^‐shielded phosphates reduce these effects. The ATP molecular structure was obtained from the Protein Data Bank (PDB) Chemical Component Dictionary, and protein structures were obtained from the PDB using entries 1AO6 and 253 L. Molecular structures were rendered in PyMOL, and the final schematic was assembled and annotated in Microsoft PowerPoint.

ATP and multivalent phosphate ions can cause basic proteins, such as lysozyme, to undergo a phenomenon known as reentrant condensation (see Fig. 2), which has been previously seen in systems with acidic proteins and trivalent and tetravalent cations [83, 84]. In studies of lysozyme [40, 69], polyvalent anions such as pyrophosphate (PP), TPP and ATP bind to positively charged surface residues, reducing electrostatic repulsion and promoting short‐range attractions, as reflected by changes in protein–protein interactions and ζ‐potential values. At intermediate concentrations, multivalent ion bridging between neighbouring proteins drives condensation, whereas at higher concentrations, continued binding leads to overcharging and resolubilisation. TPP produces a broader phase diagram than PP, consistent with its stronger cross‐bridging ability, while ATP and TPP exhibit very similar effects on protein–protein interactions and solubility. Notably, citrate does not cause condensation, indicating the importance of phosphate chemistry rather than ion valency alone. Structural and NMR analyses further showed that ATP can simultaneously interact with multiple protein molecules and mediate intermolecular bridging [40]. However, ATP, unlike TPP, can additionally induce protein crystallisation through stronger binding at the canonical nucleic‐acid‐binding region. Phase behaviour is further modulated by salts such as NaCl and MgCl_2_, which screen or disrupt ion‐bridging interactions. Although Mg–ATP retains the same local binding sites and similar apparent affinities as free ATP, Mg^2+^ complexation appears to reduce the capacity of ATP to cross‐link neighbouring protein molecules. Similar behaviour has also been observed for a mAb, where ATP induces rapid LLPS, while Mg^2+^ suppresses LLPS by chelating ATP and disrupting these interactions [30]. Similarly, Lenton et al. demonstrated that TPP induces reentrant condensation of histatin‐5 through a combination of long‐range electrostatic neutralisation and cross‐linking protein chains through specific arginine–phosphate interactions [51]. Furthermore, the addition of NaCl suppresses phase separation, with precipitation disappearing at ionic strengths comparable to those at the decondensation boundary, confirming that electrostatic screening governs the disruption of these ion‐bridging interactions.

The role of ATP in modulating LLPS of intrinsically disordered proteins (IDPs) was first clearly established by Patel et al., who described ATP as a biological hydrotrope capable of preventing and dissolving phase‐separated condensates of proteins such as FUS at physiological millimolar concentrations [3]. However, in the light of later studies, this behaviour should not be interpreted as classical hydrotropy alone, but rather as a system‐dependent combination of phosphate‐mediated electrostatics, hydration/Hofmeister effects and adenosine‐associated contacts [17, 37, 65]. ATP suppressed LLPS and resolubilised preformed droplets over a concentration range of ∼2–8 mm. GTP displayed similar droplet‐dissolving activity to ATP, suggesting that this effect is not unique to the adenine nucleobase, whereas ADP and AMP required higher concentrations, indicating that the triphosphate group makes an important contribution to solubilisation. In contrast, TPP was ineffective at resolubilisation, suggesting that ATP does not behave simply as a multivalent electrolyte in these systems, but instead requires the combined contributions of the nucleobase and triphosphate moieties.

Subsequent studies showed that LLPS in FUS is driven primarily by cation–π interactions between arginine‐rich regions and aromatic residues [24, 85]. ATP binds preferentially to Arg/Lys‐rich regions and exhibits strongly concentration‐dependent effects: low ATP concentrations (∼1–2 mm) can enhance LLPS, consistent with weak intermolecular bridging, whereas higher concentrations suppress LLPS through saturation of binding and disruption of the interaction network. Although direct ATP–aromatic interactions are not detected experimentally at low ATP concentrations, Kitamura et al. showed that ATP is substantially more effective than TPP at suppressing LLPS and aggregation of FUS, which was attributed to additional interactions involving the adenosine moiety and aromatic residues supported by molecular dynamics simulations [31]. Consistent with this, studies on CAPRIN1 showed that ATP binding at low concentrations (∼1 mm) is dominated by interactions with arginine residues that enhance LLPS through intermolecular cross‐bridging, whereas at much higher ATP concentrations (∼50 mm), additional interactions involving aromatic residues emerge and coincide with resolubilisation of the condensates [25, 27].

Mg^2+^ complexation appears to modulate ATP‐mediated LLPS of IDPs in a system‐dependent manner. Many FUS studies used Mg^2+^‐containing ATP conditions without independently varying Mg^2+^ [3]; for example, ATP was often titrated in the presence of fixed MgCl_2_, so both Mg–ATP and excess free ATP may contribute [24, 85]. Nevertheless, ATP retains concentration‐dependent effects on FUS LLPS under these conditions, and Kitamura et al. found that ATP and equimolar Mg–ATP had similar effects on FUS cloud points [31]. In contrast, Mahapatra et al. reported that ATP promoted rapid FUS droplet formation only in the presence of Mg^2+^, indicating that Mg–ATP can enhance phase separation in some contexts [82]. Similar system dependence is seen in other IDPs: Mg–ATP and Mg^2+^‐free ATP appear to produce broadly comparable reentrant behaviour in CAPRIN1 [25, 27]. Thus, Mg^2+^ can preserve, attenuate or enhance ATP‐mediated LLPS depending on the dominant interaction mechanism.

## Impact of ATP on protein aggregation and fibril formation

The effects of ATP on amyloid aggregation were first systematically examined by Patel et al., who showed that millimolar ATP suppresses fibril formation across a range of amyloidogenic systems, including the prion domain of the yeast transcription factor Mot3 and Aβ [3]. Similar ATP‐mediated suppression of fibrillation was later reported by Song and coworkers for several neurodegeneration‐associated RNA‐binding proteins, where ATP binding to the FUS and TDP‐43 RRM domains reduced amyloid formation [41, 42, 43]. These studies were generally performed under Mg^2+^‐containing ATP conditions. A particularly important observation is that ATP suppresses fibrillation of Aβ, whereas TPP enhances aggregation, a behaviour later confirmed by Mehringer et al. under Mg^2+^‐free ATP conditions [17]. In these studies, suppression of fibrillation generally occurs at relatively high ATP concentrations (∼10–20 mm), and molecular simulations suggest that ATP disrupts prefibrillar assemblies through interactions involving aromatic residues [17, 63]. Additional mechanisms emerge at lower ATP concentrations. Coskuner and Murray showed that ATP suppresses Aβ misfolding at concentrations as low as ∼0.5–1 mm through interactions involving Tyr10, demonstrating that relatively weak ATP–aromatic interactions can influence early misfolding events [60]. Kuramochi et al. further showed that ATP (∼10 mm) increases the conformational dynamics of Aβ, diverting aggregation towards off‐pathway amorphous species rather than ordered fibrils, with substantially weaker effects observed for ADP and AMP [22]. In contrast, Exley reported that ATP accelerates fibrillation of the highly amyloidogenic Aβ(25–35) fragment, highlighting that ATP effects depend strongly on peptide sequence and aggregation pathway [86].

Conversely, ATP can accelerate aggregation of strongly cationic intrinsically disordered proteins (IDPs). Heo et al. showed that both ATP and TPP strongly accelerate fibrillation of the lysine‐rich tau K18 domain and human islet amyloid polypeptide (hIAPP), whereas adenosine has little effect, indicating that the dominant interactions arise from the triphosphate group [23]. ATP promoted fibrillation over the range 0.1–10 mm yet was largely absent from the mature fibrils, suggesting a catalytic role in nucleation through transient reduction of electrostatic repulsion rather than direct incorporation into the fibril structure [23]. In contrast, Dec et al. demonstrated that an amyloidogenic insulin peptide conjugated to polylysine segments undergoes ATP‐dependent fibrillation in which ATP becomes incorporated into the fibrils at defined stoichiometries [20, 29]. In particular, fibrils formed by the conjugated peptide incorporated two ATP molecules per peptide, leading to near‐complete charge neutralisation of the K_8_ segment, while additional stabilisation was attributed to adenine stacking interactions within the fibril structure. Increasing the polylysine length shifted the aggregation pathway from direct fibrillation towards ATP‐induced LLPS followed by amyloid formation. Consistent with this behaviour, Kota et al. showed that ATP induces LLPS of highly basic lysine‐ and arginine‐rich peptides, with ATP becoming highly enriched within the condensates and stronger effects observed for arginine‐rich systems [28]. Equimolar MgCl_2_ did not abolish pK–ATP droplet formation, but only shifted the pK phase diagram, indicating that Mg–ATP retains sufficient multivalent bridging capacity [28].

Studies on α‐synuclein further illustrate how ATP modulates aggregation primarily through multivalent electrostatic interactions with positively charged residues. ATP binds weakly to the lysine/threonine‐rich N‐terminal ‘KTKEGV’ repeats, disrupting long‐range electrostatic contacts that normally shield the hydrophobic NAC region and thereby promoting early aggregation events and shortening the lag time for fibril formation [18]. This concentration‐dependent effect is retained under Mg^2+^‐containing conditions, although Mg^2+^ complexation weakens direct ATP binding to the N‐terminal region [18]. Similar behaviour was observed for inorganic polyphosphates and ATP analogues, where aggregation‐promoting effects become stronger with increasing phosphate number, emphasising the dominant role of the multivalent phosphate groups [21, 87]. At higher ATP concentrations, however, distinct mechanisms emerge: Kamski–Hennekam et al. observed suppression of late‐stage fibril growth, whereas Yamaguchi et al. attributed the second aggregation regime to Hofmeister salting‐out effects and altered solvation [18, 21, 87].

ATP has also been shown to promote aggregation of globular proteins under conditions that induce partial unfolding and expose positively charged residues. Bhattacharya et al. showed that ATP rapidly induces amyloid‐like aggregation and fibrillation of BSA and ovalbumin at pH 2, while TPP produced nearly identical effects and adenosine had little influence, demonstrating that the dominant interactions arise from the triphosphate group [19]. BSA aggregated more rapidly than ovalbumin, consistent with its higher lysine content and greater capacity for multivalent ATP binding. In contrast, lysozyme, which contains fewer positively charged residues and is relatively enriched in arginine residues, formed nonfibrillar crystalline‐like aggregates rather than extended amyloid fibrils [19]. Similarly, Calamai et al. showed that ATP strongly accelerates fibril formation of partially unfolded human lysozyme and acylphosphatase under mildly acidic and elevated‐temperature conditions, with the enhancement decreasing in the order ATP > ADP > AMP [81]. Addition of Mg^2+^ suppressed both ATP binding and aggregation enhancement, further supporting the central role of electrostatic interactions. More recently, Yamaguchi et al. reported that ATP also enhances fibril formation of insulin, *β*_2_‐microglobulin, and α‐synuclein at pH 2, where the proteins adopt molten‐globule‐like conformations [87]. The authors proposed that ATP binding reduces intramolecular electrostatic repulsion and stabilises compact partially folded intermediates that are more aggregation‐prone. Consistent with this mechanism, the aggregation‐promoting effect again scaled with phosphate number in the order ATP > ADP > AMP.

In contrast to the aggregation‐promoting effects observed for positively charged proteins under acidic conditions, our studies have shown that ATP and TPP strongly suppress aggregation of acidic proteins under near‐neutral conditions, where the proteins possess a net negative charge (see Fig. 2) [54, 55]. For ovalbumin, BSA, recombinant HSA and ribonuclease A (RNaseA), addition of ATP or TPP dramatically reduced aggregate growth rates during thermal stress, despite the proteins undergoing unfolding transitions. ATP and TPP exhibited nearly identical behaviour, indicating that the dominant interactions arise from the multivalent phosphate groups rather than the adenine moiety. ζ‐potential and protein–protein interaction measurements showed that ATP and TPP binding causes overcharging of the proteins and substantially increases intermolecular electrostatic repulsion [54, 55]. Consistent with this, aggregation kinetics shifted from rapid aggregate–aggregate condensation to slower monomer‐addition and ultimately nucleation‐dominated behaviour as ATP or TPP concentration increased. Aggregate growth rates exhibited a pronounced minimum at intermediate concentrations, reflecting the competition between preferential ion binding and electrostatic screening effects.

## Conclusions and future perspectives

Overall, the key determinants governing noncanonical ATP binding to proteins are becoming increasingly clear. ATP interacts preferentially with positively charged surface residues, particularly arginine and lysine, whereas interactions involving aromatic residues appear to be comparatively weaker and more system dependent. Rather than binding through a small number of discrete sites, ATP likely associates with protein surfaces through a dynamic ensemble of weak, rapidly exchanging interactions spanning a broad range of affinities. Significant progress has also been made in understanding how these interactions influence different forms of protein self‐assembly, although several fundamental questions remain unresolved. In particular, ATP often resembles TPP or other multivalent phosphate species, supporting a dominant role for the triphosphate group in electrostatics, hydrogen bonding, hydration, charge regulation and intermolecular bridging. However, important examples also exist where ATP and TPP diverge or even oppose one another. At present, it remains unclear whether these differences arise primarily from distinct hydration and solvation properties [88] or from the ability of the adenosine moiety to participate in additional cation–π, π–π, or nucleotide‐specific contacts with residues such as arginine and aromatic side chains. This dual character may explain why ATP behaves similar to inorganic polyphosphates in triphosphate‐dominated systems, while retaining molecule‐specific effects in Arg‐rich, aromatic‐rich or nucleic‐acid‐binding protein environments.

An important implication of these studies is that ATP should not be viewed as uniquely active in all contexts. Many of its effects can be reproduced, at least partially, by other multivalent phosphate‐containing molecules. TPP and inorganic polyphosphates mimic ATP in systems dominated by electrostatic binding, charge regulation and phosphate‐mediated bridging, while ADP and AMP often show weaker effects that scale with the number of phosphate groups. Similarly, GTP, CTP and UTP can produce ATP‐like weak binding signatures in some protein systems, and GTP can display hydrotropic activity comparable to or greater than ATP in model aromatic‐solute assays. These observations suggest that the biochemical basis of ATP‐mediated modulation is not a single ATP‐specific recognition event, but rather the combination of multivalent phosphate interactions, hydration and solvation effects, cation binding, and weaker nucleobase‐associated contacts. Nevertheless, ATP is uniquely positioned among cellular metabolites because it combines these physicochemical properties with unusually high intracellular abundance, and strong coupling to Mg^2+^ speciation. Thus, rather than acting as a uniquely specialised proteostasis factor, ATP may represent the most abundant member of a broader class of small, concentrated, amphiphilic and highly charged metabolites capable of modulating protein solubility, conformational stability, and self‐assembly. Establishing whether these effects constitute an evolved biochemical function, or instead an emergent consequence of maintaining millimolar nucleotide pools, remains an important open question.

Addressing these questions will require the integration of high‐resolution experimental measurements with molecular simulations. While molecular simulations have provided valuable insights into ATP–protein interactions [57, 58, 61, 62, 63, 65], they must be interpreted cautiously, as accurate force fields for ATP and other highly charged polyphosphates are still under active development [17, 65, 89]. In particular, nonpolarisable water models can exaggerate electrostatic interactions and promote artificial ATP clustering [90, 91]. A further challenge is translating mechanisms established *in vitro*, particularly those based on free ATP, to cellular environments. Because intracellular ATP is extensively partitioned between free ATP, Mg–ATP, and other ligand‐bound states across heterogeneous microenvironments, local Mg^2+^ availability, ionic strength, pH, and biomolecular compartmentalisation are likely to determine whether ATP behaves predominantly as a phosphate‐mediated electrostatic modulator, a weak nucleotide‐like ligand, or a broader regulator of protein solubility and self‐assembly.

## Conflict of interest

The authors declare no conflict of interest.

## Author contributions

ST performed the literature review, analysed and interpreted the relevant studies, and wrote the initial manuscript draft. RC contributed to the conceptual development of the review, provided supervision and critically revised the manuscript.
